# Supplementary material for: Accuracy of a Prehospital Triage Protocol in Predicting In-Hospital Mortality and Severe Trauma Cases among Older Adults
Source: Int J Environ Res Public Health. 2023 Jan 20;20(3):1975. doi: 10.3390/ijerph20031975 (PMC9916137; doi:10.3390/ijerph20031975)
Supplement: Supplementary file 1 [file ijerph-20-01975-s001.zip › Table S1.pdf]

Table S1: Older adult characteristics according to age subgroups.

	65-74 years n=638	75-84 years n=432	≥85 years n=180	p-value
Age	69 [66;72]	79 [77;82]	87 [86;90]	**<0.0001**
Sex, male	442/633 (69.8)	256/430 (59.5)	93/180 (51.7)	**<0.0001**
Type of trauma				0.4497
Blunt	580/630 (92.1)	6,823 (90.2)	1,136 (92.3)	
Penetrating	50/630 (7.9)	28/423 (6.6)	17/178 (9.6)	
Mechanism				0.366
Road collision	315/632 (49.8)	4208/425 (48.9)	85/178 (47.8)	
Car occupant	139/315 (44.1)	105/208 (50.5)	40/85 (47.1)	
Pedestrian	73/315 (23.2)	74/208 (35.6)	37/85 (43.5)	
Bicycle	70/315 (22.2)	25/208 (12.0)	5/85 (5.9)	
Motorcyclist	30/315 (9.5)	4/208 (1.9)	2/85 (2.4)	
Fall	219/632 (34.7)	157/425 (36.9)	68/178 (38.2)	
Gunshot, stab	34/632 (5.4)	22/425 (5.2)	15/178 (8.4)	
Other	64/632 (10.1)	38/425 (8.9)	10/178 (5.6)	
Delays				
On-scene time	34 [25;45]	35 [25;47]	35 [25;48]	0.41
Total prehospital time[a]	87 [65;115]	85 [64;110]	84 [65;105]	0.4002
Prehospital medical evaluation				
Systolic blood pressure	138 [119;160]	143 [120;166]	142 [122;171]	**0.0368**
Heart rate	81 [70;95]	84 [69;97]	82 [70;95]	0.7175
SpO2 <95%	1141/520 (27.1)	109/349 (31.2)	49/151 (32.5)	0.2795
Hemoglobin <9 g/dL	14/516 (2.7)	18/349 (5.2)	11/141 (7.8)	**0.0181**
Shock index >1	35/556 (6.3)	28/387 (7.2)	10/159 (6.3)	0.8354
GCS score ≤8	102/578 (17.7)	74/395 (18.7)	41/168 (24.4)	0.1429
Prehospital ALS procedures				
Mechanical ventilation	146/561 (26.0)	116/375 (30.9)	45/160 (28.1)	0.261
Thoracostomy	4/558 (0.7)	4/371 (1.1)	1/157 (0.6)	0.8041
Packed red blood cell transfusion	9/569 (1.6)	9/384 (2.3)	3/161 (1.9)	0.6976
Fluid resuscitation	437/572 (76.4)	301/385 (78.2)	128/162 (79.0)	0.7037
Vasopressor infusion	69/569 (12.1)	51/384 (13.3)	20/161 (12.4)	0.8686
Tranexamic acid	255/494 (51.6)	189/328 (57.6)	77/146 (52.7)	0.2301
Pelvic binder	67/555 (12.1)	46/372 (12.4)	12/157 (7.6)	0.2542
FAST	160/550 (29.1)	88/363 (24.2)	47/158 (29.8)	0.2202
Anticoagulant and/or antiplatelet medication	165/594 (27.8)	177/411 (43.1)	95/172 (55.2)	**<0.0001**
Severity grade				0.1742
A	88/638 (13.8)	64/432 (14.8)	38/180 (21.1)	
B	177/638 (27.7)	126/432 (29.2)	46/180 (25.6)	
C	373/638 (58.5)	242/432 (56.0)	96/180 (53.3)	

Data are presented as median [IQR] or n (%); proportions (%) were calculated among those with data

[a]From call to hospital arrival, expressed in minutes

ALS: Advanced life support; FAST: Focused assessment with sonography for trauma; GCS: Glasgow coma scale; SpO2: Peripheral oxygen saturation measured by pulse oximetry
