# Supplementary material for: Accuracy of a Prehospital Triage Protocol in Predicting In-Hospital Mortality and Severe Trauma Cases among Older Adults
Source: Int J Environ Res Public Health. 2023 Jan 20;20(3):1975. doi: 10.3390/ijerph20031975 (PMC9916137; doi:10.3390/ijerph20031975)
Supplement: Supplementary file 1 [file ijerph-20-01975-s001.zip › Table S2.pdf]

Table S2: Performance of the triage protocol to detect patients who died during their in-hospital stay or with a severe trauma among older patients groups.

		In-hospital mortality				ISS > 15			
		**65-74 years**	**75-84 years**	**≥85 years**	**p-value**	**65-74 years**	**75-84 years**	**≥85 years**	**p-value**
Grade A	Se	56.2 [44.7;67.3]	42.9 [32.5;53.7]	55.1 [40.2;69.3]	0.1653	22.0 [17.8;26.8]	22.5 [17.5;28.2]	29.8 [21.6;39.1]	0.2136
	Sp	93.2 [90.6;95.3]	94.5 [91.2;96.8]	91.5 [85.0;95.9]	0.5406	95.9 [93.0;97.9]	96.1 [92.1;98.4]	93.9 [85.2;98.3]	0.7425
	PPV	57.7 [46.0;68.8]	70.9 [57.1;82.4]	73.0 [55.9;86.2]	0.1559	86.4 [77.4;92.8]	89.1 [78.8;95.5]	89.5 [75.2;97.1]	0.8332
	NPV	92.8 [90.2;95.0]	84.0 [79.6;87.8]	83.1 [75.5;89.1]	**0.0001**	51.1 [46.8;55.3]	46.7 [41.5;52.0]	43.7 [35.4;52.2]	0.1957
Grade B	Se	91.2 [82.8;96.4]	89.0 [80.7;94.6]	87.8 [75.2;95.4]	0.8001	59.1 [53.7;64.4]	62.1 [55.8;68.1]	58.8 [49.2;67.9]	0.7336
	Sp	67.7 [63.3;71.8]	72.0 [66.4;77.1]	70.3 [61.2;78.4]	0.4460	79.2 [74.1;83.7]	81.6 [75.1;87.0]	74.2 [62.0;84.2]	0.4510
	PPV	31.7 [25.8;38.2]	50.0 [42.1;57.9]	55.1 [43.4;66.4]	**0.0001**	77.0 [71.4;81.9]	82.6 [76.5;87.7]	79.8 [69.6;87.7]	0.3382
	NPV	97.9 [95.8;99.2]	95.4 [91.7;97.8]	93.3 [85.9;97.5]	0.0671	62.2 [57.1;67.1]	60.3 [53.9;66.5]	51.0 [40.6;61.4]	0.1372
