# Supplementary figures and images for: Accuracy of a Prehospital Triage Protocol in Predicting In-Hospital Mortality and Severe Trauma Cases among Older Adults
Source: Int J Environ Res Public Health. 2023 Jan 20;20(3):1975. doi: 10.3390/ijerph20031975 (PMC9916137; doi:10.3390/ijerph20031975)

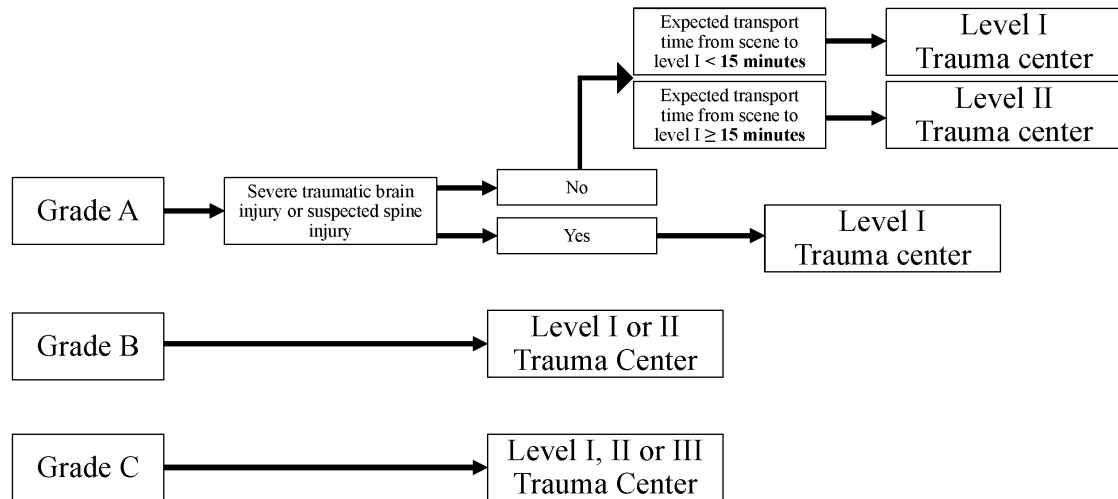

**Figure S1:** Triage protocol.

Supplement: Supplementary file 1 [file ijerph-20-01975-s001.zip › Figure S1.pdf]

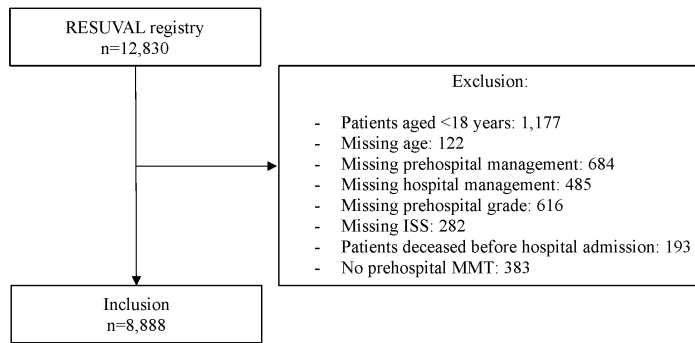

**Figure S2:** Flow chart.

Supplement: Supplementary file 1 [file ijerph-20-01975-s001.zip › Figure S2.pdf]
